# Sub-optimal cholesterol response to initiation of statins and future risk of cardiovascular disease

**DOI:** 10.1136/heartjnl-2018-314253

**Published:** 2019-04-15

**Authors:** Ralph Kwame Akyea, Joe Kai, Nadeem Qureshi, Barbara Iyen, Stephen F Weng

**Affiliations:** Division of Primary Care, University of Nottingham, Nottingham, UK

**Keywords:** lipoproteins and hyperlipidemia, epidemiology, electronic medical records

## Abstract

**Objective:**

To assess low-density lipoprotein cholesterol (LDL-C) response in patients after initiation of statins, and future risk of cardiovascular disease (CVD).

**Methods:**

Prospective cohort study of 165 411 primary care patients, from the UK Clinical Practice Research Datalink, who were free of CVD before statin initiation, and had at least one pre-treatment LDL-C within 12 months before, and one post-treatment LDL-C within 24 months after, statin initiation. Based on current national guidelines, <40% reduction in baseline LDL-C within 24 months was classified as a sub-optimal statin response. Cox proportional regression and competing-risks survival regression models were used to determine adjusted hazard ratios (HRs) and sub-HRs for incident CVD outcomes for LDL-C response to statins.

**Results:**

84 609 (51.2%) patients had a sub-optimal LDL-C response to initiated statin therapy within 24 months. During 1 077 299 person-years of follow-up (median follow-up 6.2 years), there were 22 798 CVD events (12 142 in sub-optimal responders and 10 656 in optimal responders). In sub-optimal responders, compared with optimal responders, the HR for incident CVD was 1.17 (95% CI 1.13 to 1.20) and 1.22 (95% CI 1.19 to 1.25) after adjusting for age and baseline untreated LDL-C. Considering competing risks resulted in lower but similar sub-HRs for both unadjusted (1.13, 95% CI 1.10 to 1.16) and adjusted (1.19, 95% CI 1.16 to 1.23) cumulative incidence function of CVD.

**Conclusions:**

Optimal lowering of LDL-C is not achieved within 2 years in over half of patients in the general population initiated on statin therapy, and these patients will experience significantly increased risk of future CVD.

## Introduction

Cardiovascular disease (CVD) remains the leading cause of death globally.^1^ The positive correlation between the incidence of CVD and levels of low-density lipoprotein cholesterol (LDL-C) concentration has been well-established.^2^ Statins are recognised as being effective in lowering cholesterol and reducing the risk of future CVD events for both primary or secondary prevention.^3^

Following a meta-analysis of cholesterol treatment trials,^4^ the percentage reductions in LDL-C achieved by specific statins (and their doses) have been established from the data of patients in whom statins reduced CVD events. Accordingly, national guidelines in the USA and UK recommend intended LDL-C reduction targets for statin therapy to reduce CVD.^5 6^ The 2013 American College of Cardiology/American Heart Association (ACC/AHA) guidelines suggested a fixed dose (or intensity) of statin for each risk category, with an intended LDL-C reduction of 30–49% and ≥50% for moderate and high intensity statins, respectively.^6^ The National Institute for Health and Care Excellence (NICE) guidelines in the UK aim for >40% reduction in non-HDL-C.^5^

Both individual biological and genetic variability in LDL-C response to statin therapy,^7^ as well as variation in adherence, have been identified.^8^ However, there is limited evidence on variation in LDL-C response in the general population for patients initiated on statins for primary prevention of CVD.

In this large, prospective open population cohort study, we sought to assess differences in LDL-C response in primary care patients initiated on statins and their impact on future CVD events.

## Methods

### Data source

This prospective cohort study used the UK Clinical Practice Research Datalink (CPRD) of primary care electronic health records, linked to Hospital Episode Statistics (HES) and Office for National Statistics (ONS) data. HES provides information on all hospital admissions and the ONS records cause-specific mortality for all deaths in England and Wales. The CPRD database contains anonymised patient data from 681 family physician practices (approximately 8% of the UK population) and is broadly representative of the general population in terms of age, sex and ethnicity. CPRD has good ascertainment of major diagnoses such as hypertension and other chronic conditions, and has provided a key source of evidence for major research.^9^ This study was approved by the Independent Scientific Advisory Committee for the Medicines and Healthcare Products Regulatory Agency database research (ISAC Protocol 17_200R).

### Study population

This cohort study comprised 183 213 patients (males and females) initiated on statin therapy between 3 September 1990 and 7 June 2016 identified from the CPRD database. Patients were eligible for inclusion if they were registered with their family practice for at least 12 months, and their electronic health records met CPRD quality control.^9^ Patients had at least two recorded LDL-C measurements (at least one measurement within 12 months before statin initiation and one measurement within 24 months after statin initiation). All individuals who had a CVD event any time before initiating statin therapy were excluded.

### Exposure

Based on current NICE Cardiovascular Disease Guidelines (CG181),^5^ patients on treatment with statins for primary prevention of CVD who failed to achieve >40% reduction in untreated baseline LDL-C record within the first 24 months were defined as sub-optimal responders. The untreated baseline LDL-C record used was the most recent within 12 months before or at the initiation of statin therapy. For post-treatment response, the most recent LDL-C record at or closest to 24 months was used. While a 12 month follow-up period to gauge treatment response is recommended by national guidelines, 24 months was considered a more pragmatic timeframe to ascertain post-treatment LDL-C in this study. In general practice there is typically slippage on the 12 months follow-up cholesterol test and patient review. The 24 month time point ensures all follow-up cholesterols are captured.

### Outcomes

CVD events were defined as: coronary heart disease (CHD), stroke/transient ischaemic attack (TIA), peripheral vascular disease (PVD), or CVD-related death. Follow-up for incident CVD was from 24 months after first prescription of any statin, to first record of either a CVD event, non-CVD-related death, transfer out of the practice, or the end of CPRD data collection. Patients with a recorded CVD event within the 24 month exposure period were excluded from the analyses.

Evidence from both randomised trials and cohort studies show the full effect of serum cholesterol reduction on risk of CVD is not achieved until after the first 2 years of statin initiation.^10^ Therefore, any inclusion of CVD outcome events in the first 2 years (before the full effect of reducing serum LDL-C concentration is achieved) would underestimate the full effect.^3^ Thus, individuals with CVD events in the first 2 years (our defined exposure period) were excluded from analysis to avoid underestimation of our results.

CVD events were identified from primary and secondary care records and the ONS death registry. In CPRD, ‘medcodes’ are mapped equivalents of the widely used Read clinical coding system in primary care in the UK. Codes for CVD events in CPRD have been reported previously.^11^ International Classification of Diseases, 10th revision (ICD-10) codes were used to identify CVD-related events in HES and ONS. In HES and ONS, a CVD event was recorded if it was coded as the primary diagnosis across a hospitalisation or the underlying cause of death, respectively.

### Baseline cardiovascular risk factors

We assessed baseline covariates (online supplementary appendix 1) as potential confounders for the relation between statin therapy response and future CVD event. The most recent values within 12 months before the study entry date (first prescribed statin date) were used for smoking status, alcohol misuse, systolic and diastolic blood pressures, prescription of medications and medication count at baseline. Year of statin initiation (accounting for time differences), the number of LDL-C measurements recorded (accounting for frequency of monitoring) and change of statin potency within the exposure period were also considered as potential confounders. All other covariates were based on the latest record before the study entry date. Deprivation was based on the English Index of Multiple Deprivation 2015 (IMD)^12^ linked to patient postcode, with patients grouped by fifths. The IMD is a weighted aggregate for the local neighbourhood (mean population 1500) of deprivation across seven domains: income; employment; health deprivation and disability; education, skills, and training; barriers to housing and services; living environment; and crime.

10.1136/heartjnl-2018-314253.supp1 Supplementary file 1

### Statistical analysis

To obtain estimates of the association between sub-optimal cholesterol response to statin therapy and the incidence of CVD in our cohort, we performed Cox proportional hazards regression analysis, adjusting for significant baseline covariates. In this analysis, informative censoring of the survival time was taken into account by considering patients transferring out of the practice and death as competing risks for incident CVD. We therefore performed a competing-risk analysis which provided the cause-specific (or sub-) hazard ratio (HR) and used it to calculate the cumulative incidence of the CVD outcomes of interest. We similarly adjusted for significant covariates for the competing-risks regression based on Fine and Grey’s^13^ proportional sub-hazards model.

We carried out a sensitivity analysis restricting the cohort to only patients with post-treatment LDL-C after at least 3 months of statin initiation (in line with the NICE lipid guideline recommendation of first monitoring LDL-C at 3 months from statin initiation).

Multiple imputation by chained equations procedure in Stata^14^ was used to estimate missing values for body mass index (BMI) and systolic and diastolic blood pressures. All other patient variables were included in the imputation models to create 10 imputed datasets. The change-in-estimate criterion^15^ was used to determine significant covariates. Covariates were considered significant if they altered the unadjusted exposure-outcome effect by 5% or more.

Further analysis was performed to assess the relationship between LDL-C reduction as a continuous variable and the risk of CVD in the population of patients who had a reduction in LDL-C over the follow-up period.

All statistical analyses were performed using Stata 15.1 (StataCorp LP). Values of p<0.05 were considered to be statistically significant.

## Results

From the 207 230 patients identified from CPRD, 17 872 patients were excluded due to having a prior record of a CVD event. There were 23 947 patients with a CVD event within 24 months of initiating therapy (exposure period) and hence they were excluded from the analysis (online supplementary appendix 2). The cohort, therefore, included 165 411 eligible patients initiating statin therapy. There were 84 609 (51.2%) patients with a sub-optimal LDL-C response at 24 months after initiating statin therapy. The mean age at therapy initiation was 62.4 years and 48.6% of the study population were women. The median follow-up time was 5.9 and 6.5 years for sub-optimal and optimal statin responders, respectively. A higher proportion of patients with a sub-optimal response were prescribed lower potency statins compared with those with an optimal response. There were differences between groups by age, gender, deprivation, smoking status, baseline LDL-C and statin potency (p<0.05). Table 1 shows the baseline characteristics of the entire cohort and online supplementary appendix 3 shows the baseline characteristics stratified by year of statin initiation.

**Table 1:** Characteristics of eligible patients being treated with statins and free from cardiovascular disease at baseline. Patients are stratified by response to statin (based on LDL-C measurement) 24 months after initiation of statin therapy

Characteristics		Total number (%)	Optimal statin responders, n (%)	Sub-optimal statin responders, n (%)
		165 411 (100)	80 802 (48.85)	84 609 (51.15)
Follow-up time (years)	Median (IQR)	6.2 (3.4–9.3)	6.5 (3.6–9.6)	5.9 (3.3–9.1)
Females	No. (%)	80 370 (48.6)	40 739 (50.4)	39 631 (46.8)
Age (years)	Mean (SD)	62.4 (11.8)*	63.6 (11.4)	61.3 (12.0)
Baseline LDL-C (mmol/L)	Mean (SD)	4.1 (1.1)	4.4 (1.1)	3.8 (1.1)
Post-treatment LDL-C within 24 months (mmol/L)	Mean (SD)	2.6 (1.0)	2.1 (0.6)	3.1 (1.0)
BMI (kg/m^2^)	Mean (SD)	29.1 (5.7)†	29.0 (5.5)	29.3 (5.9)
Systolic blood pressure (mm Hg)	Mean (SD)	143 (19)‡	144 (20)	141 (19)
Diastolic blood pressure (mm Hg)	Mean (SD)	83 (11)§	83 (11)	82 (11)
Alcohol misuse	No. (%)	1231 (0.7)	493 (0.6)	738 (0.9)
Smoking				
Non-smoker	No. (%)	2561 (1.6)	1259 (1.6)	1302 (1.5)
Ex-smoker		1838 (1.1)	881 (1.1)	957 (1.1)
Smoker		2003 (1.2)	868 (1.1)	1135 (1.3)
Unknown status		159 009 (96.1)	77 794 (96.3)	81 215 (96.0)
Index of Multiple Deprivation (patients)				
1	No. (%)	21 945 (22.9)	11 044 (23.8)	10 901 (22.1)
2		21 142 (22.1)	10 467 (22.6)	10 675 (21.6)
3		19 224 (20.1)	9288 (20.0)	9936 (20.1)
4		18 186 (19.0)	8583 (18.5)	9603 (19.4)
5		15 291 (16.0)	6990 (15.1)	8301 (16.8)
**Comorbidities (before first statin)**				
Diabetes				
Non-diabetic	No. (%)	137 984 (83.42)	67 739 (83.8)	70 245 (83.0)
Poorly-controlled diabetic		9246 (5.6)	4351 (5.4)	4895 (5.8)
Well-controlled diabetic		3253 (2.0)	1698 (2.1)	1555 (1.8)
Diabetic control status unknown		14 928 (9.0)	7014 (8.7)	7914 (9.4)
Dyslipidaemias	No. (%)	13 445 (8.1)	6351 (7.9)	7094 (8.4)
Atrial fibrillation	No. (%)	4849 (2.9)	2473 (3.1)	2376 (2.8)
Chronic kidney disease	No. (%)	4469 (2.7)	2264 (2.8)	2205 (2.6)
Family history of cardiovascular disease	No. (%)	17 522 (10.6)	8495 (10.5)	9027 (10.7)
Family history of hyperlipidaemia	No. (%)	389 (0.2)	183 (0.2)	206 (0.2)
Treated hypertension	No. (%)	43 242 (26.1)	22 711 (28.1)	20 531 (24.3)
Hypothyroidism	No. (%)	6867 (4.2)	3492 (4.3)	3375 (4.0)
Liver disease	No. (%)	1556 (0.9)	729 (0.9)	827 (1.0)
Migraine	No. (%)	3612 (2.2)	1800 (2.2)	1812 (2.1)
Nephrotic syndrome	No. (%)	111 (0.1)	50 (0.1)	61 (0.1)
Rheumatoid arthritis	No. (%)	1465 (0.9)	718 (0.9)	747 (0.9)
Severe mental illness	No. (%)	710 (0.4)	350 (0.4)	360 (0.4)
Systemic lupus erythematosus	No. (%)	181 (0.1)	77 (0.1)	104 (0.1)
**Medications (prescribed within 12 months before first statin)**				
Medication count	Median (IQR)	6 (3–9)	6 (3–9)	6 (3–9)
Antipsychotics	No. (%)	6919 (4.2)	3634 (4.5)	3285 (3.9)
Other lipid lowering medication	No. (%)	670 (0.4)	244 (0.3)	426 (0.5)
Oral corticosteroids	No. (%)	6760 (4.1)	3193 (4.0)	3567 (4.2)
Potency of initial statin prescribed				
Low	No. (%)	39 343 (23.8)	14 674 (18.2)	24 669 (29.2)
Medium		117 264 (70.9)	61 511 (76.1)	55 753 (65.9)
High		8804 (5.3)	4617 (5.7)	4187 (5.0)
**Missing data**				
Missing BMI	No. (%)	73 801 (44.6)	37 132 (46.0)	36 669 (43.3)
Missing systolic blood pressure	No. (%)	19 383 (11.7)	8357 (10.3)	11 026 (13.0)
Missing diastolic blood pressure	No. (%)	19 322 (11.7)	8317 (10.3)	11 005 (13.0)

*Mean (SD) for all 165 411 patients.

†Mean (SD) for 91 610 (optimal: 43 670; sub-optimal: 47 940) patients with baseline body mass index record.

‡Mean (SD) for 146 028 (optimal: 72 445; sub-optimal: 73 583) patients with baseline systolic blood pressure record.

§Mean (SD) for 146 089 (optimal: 72 485; sub-optimal: 73 604) patients with baseline diastolic blood pressure record.

BMI, body mass index; LDL-C, low-density lipoprotein cholesterol.

### Incident cardiovascular events

**Figure 1:**
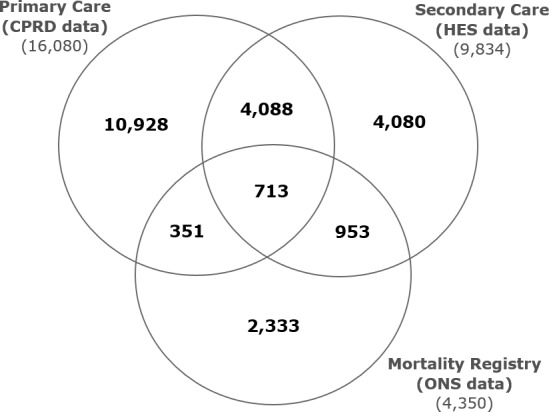
Venn diagram showing the incident CVD events recorded in each database used to ascertain CVD outcomes: primary care data (CPRD) 16 080; secondary care data (HES) 9834; mortality registry (ONS) 4350. The overlap of incident CVD events in the three datasets are also indicated: 713 in all three databases. CVD, cardiovascular disease; CPRD, Clinical Practice Research Datalink; HES, Hospital Episode Statistics; ONS, Office for National Statistics.

Combining primary care data from CPRD, mortality data from ONS and hospitalisation data from HES, incident CVD events were recorded in 22 798 (13.8%) patients: coronary artery disease, 13 142 (8.0%); stroke/TIA, 4865 (2.9%); PVD, 3097 (1.9%); CVD-related death, 1694 (1.0%). Incident CVD events were recorded exclusively in CPRD in 10 928 (6.6%) of patients. An additional 4080 (2.5%) and 2333 (1.4%) incident events were obtained from linkages to HES and ONS datasets, respectively. Some incident CVD events were recorded in more than one database (figure 1), with an overlap for all three linked databases in 713 (0.4%) patients.

### Overall incidence of cardiovascular events

**Figure 2:**
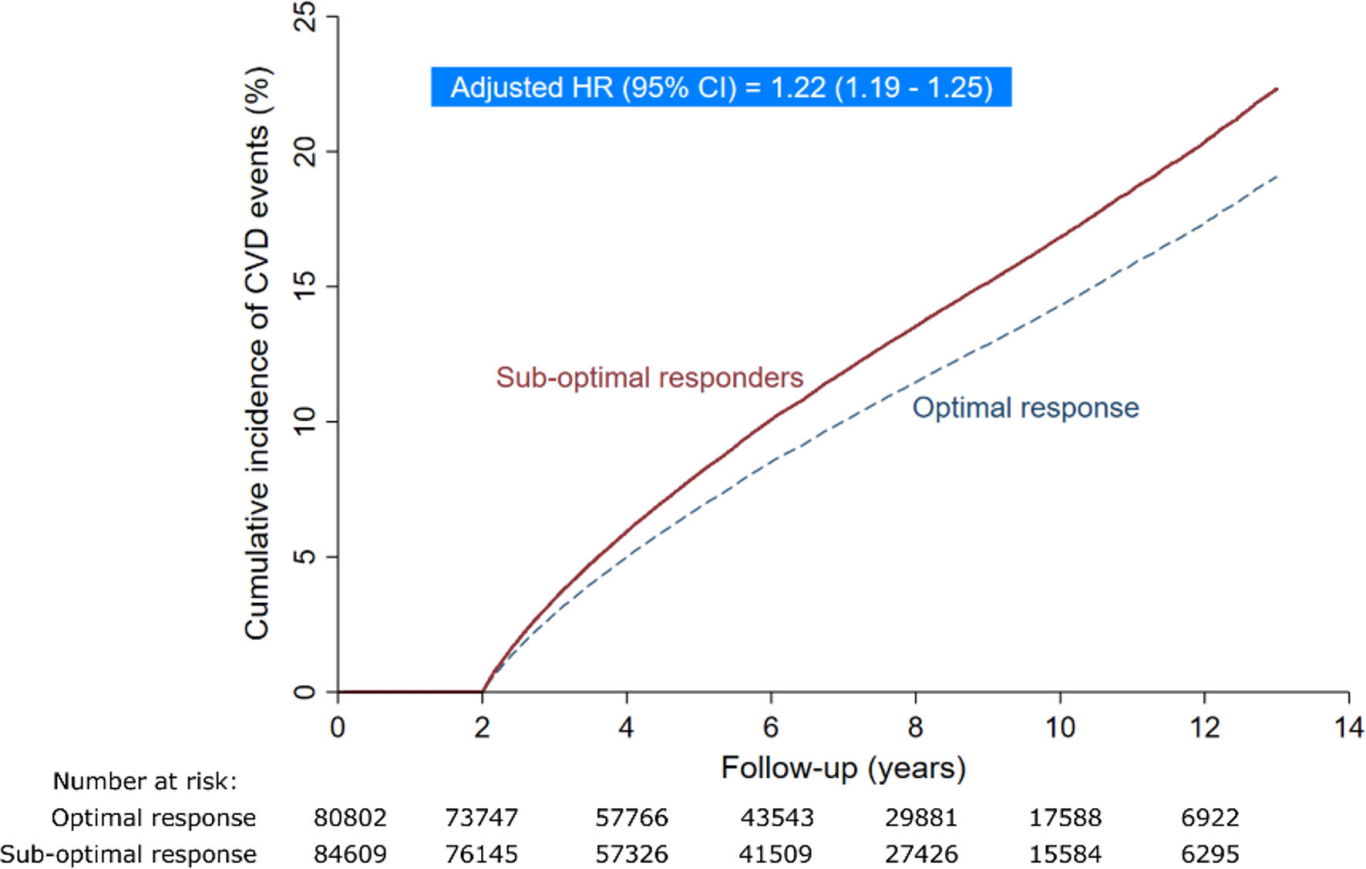
The cumulative incidence curve demonstrated that patients with a sub-optimal LDL-C response to statin therapy were associated with a higher risk of CVD events than patients with an optimal response during the follow-up period, with an adjusted HR of 1.22 (95% CI 1.19 to 1.25). Adjusted for age and baseline LDL-cholesterol level. CVD, cardiovascular disease; HR, hazard ratio; LDL-C, low-density lipoprotein cholesterol.

During 1 077 299 person-years of follow-up, there were 22 798 CVD events (12 142 in sub-optimal responders and 10 656 in optimal responders). The rate of CVD was 22.6 and 19.7 per 1000 person-years for sub-optimal and optimal responders, respectively. Patients with a sub-optimal response, compared to  those with an optimal response, were significantly more likely to have an incident CVD event (crude HR 1.17, 95% CI 1.13 to 1.20; p<0.001). After adjusting for age and baseline untreated LDL-C value, the risk of CVD remained significantly greater in sub-optimal responders compared with optimal responders (adjusted HR 1.22, 95% CI 1.19 to 1.25; p<0.001). A competing risk approach resulted in lower but similar sub-HRs for incidence of CVD for both unadjusted (1.13, 95% CI 1.10 to 1.16; p<0.001) and adjusted (1.19, 95% CI 1.16 to 1.23; p<0.001) cumulative incidence function, as shown in figure 2.

### Incidence of constituent CVD outcomes

There was a significantly increased risk of coronary artery disease, PVD and CVD-related death in patients with a sub-optimal response compared with those with an optimal response in the unadjusted models using both Cox regression and competing risk analysis. In the adjusted models, the increased risk remained significant for all the outcomes including stroke/TIA using both approaches. Although the competing risk approach resulted in lower estimates of incidence/risk, the adjusted analysis remained significant (table 2). A total of 15 630 patients had a post-treatment LDL-C record within 3 months of statin initiation. When these patients were excluded (in a sensitivity analysis – online supplementary appendix 4), the incidence of overall CVD and constituents of CVD remained significant with similar incidence rates.

**Table 2:** Effect estimates for association between sub-optimal LDL-C response at 24 months to initiated statin therapy and the risk of incident cardiovascular events using different statistical approaches (n=165 411)

	Group	Number of CVD events	Rate of CVD events (per 1000 person-years)	Crude/unadjusted models		Adjusted models	
				Cox regression	Competing-risks survival regression	Cox regression	Competing-risks survival regression
				HR (95% CI)	sHR (95% CI)	HR (95% CI)	sHR (95% CI)
Overall CVD-related event*	Optimal	10 656	19.7	1	1	1	1
	Sub-optimal	12 142	22.6	1.17 (1.13 to 1.20)	1.13 (1.10 to 1.16)	1.22 (1.19 to 1.25)	1.19 (1.16 to 1.23)†
CAD‡	Optimal	5986	11.1	1	1	1	1
	Sub-optimal	7156	13.3	1.22 (1.17 to 1.26)	1.18 (1.14 to 1.22)	1.30 (1.25 to 1.34)	1.23 (1.19 to 1.27) §
Stroke/TIA*	Optimal	2387	4.4	1	1	1	1
	Sub-optimal	2478	4.6	1.07 (1.01 to 1.13)	1.02 (0.96 to 1.08)	1.15 (1.08 to 1.22)	1.10 (1.04 to 1.17)¶
PVD*	Optimal	1462	2.7	1	1	1	1
	Sub-optimal	1635	3.0	1.14 (1.07 to 1.23)	1.09 (1.02 to 1.17)	1.16 (1.08 to 1.25)	1.12 (1.04 to 1.21)**
CVD-related death*	Optimal	821	1.5	1	1	1	1
	Sub-optimal	873	1.6	1.09 (0.99 to 1.20)	1.04 (0.95 to 1.15)	1.25 (1.13 to 1.38)	1.21 (1.09 to 1.34)††

Cox regression provides hazard ratio (HR) whereas competing-risks survival regression (Fine-Grey model) provides sub-hazard ratio (sHR).

*The multivariable Cox and competing-risk regression models for overall CVD-related events, stroke/TIA, PVD and CVD-related deaths were adjusted for age and baseline LDL-C value.

†Competing risks for overall CVD-related event were non-CVD-related death and transfer out of practice.

‡The multivariable Cox and competing-risk regression models for CAD were adjusted for age.

§Competing risks for CAD model were death, transfer out of practice, stroke/TIA and PVD.

¶Competing risks for stroke/TIA model were death, transfer out of practice, CAD and PVD.

**Competing risks for PVD model were death, transfer out of practice, CAD and stroke/TIA.

††Competing risks for CVD-related death were non-CVD-related death, transfer out of practice, CAD, stroke/TIA and PVD.

CAD, coronary artery disease; CPRD, Clinical Practice Research Datalink; CVD, cardiovascular disease; HES, Hospital Episodes Statistics, LDL-C, low-density lipoprotein cholesterol; ONS, Office of National Statistics; PVD, peripheral vascular disease; TIA, transient ischaemic attack.

In patients with a reduction in LDL-C within 24 months (n=146 355), 65 862 (45%) had a sub-optimal LDL-C response (online supplementary appendix 5). A unit reduction of 1 mmol/L LDL-C was found to be associated with a significant decrease in any CVD (OR 0.94, 95% CI 0.91 to 0.98) in patients with sub-optimal decreases in LDL-C. In this group, the decreased risk remained significant for only stroke/TIA and was not significant for other constituent CVD outcomes.

However, in patients with an optimal response, an even greater protective effect of LDL-C reduction and future CVD was seen (OR 0.87, 95% CI 0.84 to 0.90). The decreased risk remained significant for all constituent CVD outcomes.

## Discussion

### Principal findings

This large prospective population cohort study has found that over half of patients started on statin therapy for primary prevention of CVD did not experience an optimal therapeutic reduction of their LDL-C 24 months after therapy initiation. Moreover, taking into account age and baseline LDL-C values, the incidence and risk of future CVD (coronary artery disease, stroke/TIA, PVD and CVD-related death) in these patients was significantly greater compared to those with an optimal therapeutic response. The study also highlights the benefit of reducing LDL-C to optimal values, which would lead to better CVD outcomes for patients currently on statins.

### Comparison with other literature/studies

Our present findings, at a general population level, are consistent with inter-individual variation in response to statin therapy found in a smaller cross-sectional study of 22 063 patients, with up to a half (48.2%) of those prescribed statins not achieving their lipid reduction goals.^16^ As a result, despite statin therapy, many individuals remain at a much higher risk of atherosclerotic CVD, as shown in our study. Multiple patient characteristics, including sex, age, smoking status, body weight, diet and physical activity, have been reported to contribute to variations in statin-induced LDL-C reduction, but the impact of these factors is considered to be modest.^17 18^ However, variations in individual patient genotypes,^7^ and probably non-adherence,^19^ may be an important explanation for this phenomenon.

A number of cross-sectional (single-point) observational studies have investigated the prevalence of lipid disorders and statin use.^20 21^ However, these studies have used differing methodologies and data collection systems, or have been limited to specific populations (inherited lipid disorders such as familial hypercholesterolaemia) and thus their findings are not generalisable to the general population. In addition, the association between the inter-individual variations and future CVD outcomes were not explored.^20^

Meta-analysis of trial data supports the results of our study in suggesting that achieving optimal LDL-C reduction can provide cardiovascular benefits.^22^ However, the results from controlled trial settings cannot necessarily be readily extrapolated to treatment experience for patients in routine clinical practice.

Given that success in improving healthcare delivery is in part dependent on accurate estimation and reporting of the incidence and risk of various outcomes, it is imperative that future studies adequately account for the competing risks in similar analyses. For example, in a study using data from the European Renal Association-European Dialysis and Transplant Association (ERA-EDTA) Registry, the Kaplan-Meier method, compared with the competing risk method, overestimated the probabilities of the study outcomes at both 2 and 5 years' follow-up.^23^

### Clinical implications

Statins rank among the most commonly prescribed drug classes. Recent revisions of clinical guidelines for CVD risk reduction have significantly expanded the population of patients deemed eligible for statin therapy.^5 6 24^ As the high levels of statin prescribing in the UK are similar to those in the USA and many other countries,^25^ the findings should translate well, particularly to other similar western populations. There was a substantial duration of follow-up for outcomes. A wide range of potential confounders were also evaluated and adjusted for in the analyses.

Based on evidence from clinical trial settings, the response of patients to statins varies widely, with reductions in LDL-C values following the administration of statins ranging from 5–70%.^26^ In our study using a large general population reflecting routine practice, over half of the patients had a sub-optimal LDL-C response.

Currently, there is no management strategy in clinical practice which takes into account patient variations in LDL-C response,^27^ and no guidelines for predictive screening before commencement of statin therapy. Validated clinical decision tools which can predict cholesterol response to statins, or to non-statin drugs, with interventions to help clinicians to tailor and optimise statin treatments for individual patients are needed.

### Strength and limitations

To our knowledge, this study is the first large prospective general population study to quantify the variability in LDL-C response to statins and its impact on future CVD events. Strengths of the study include its prospective design, large sample size, use of multiple data linkages to maximise ascertainment of all CVD outcomes, and methods of analysis which account for competing risk events.^28^ The cohort was derived from high quality primary and secondary care care databases^9^ which are broadly representative of the general UK population. These characteristics enhance the external validity and generalisability of our findings. Future research should look to validate or replicate our findings in other databases or other populations.

Some limitations of this study are recognised. Despite investigating the potential effects of a wide range of confounders in this study, it is recognised that the use of CPRD, in common with other large population database research, precludes capture of all possible confounders. The effect of patient non-adherence to therapy could not be accounted for, but the findings nevertheless reflect real-world experience of treatment responses and outcomes for a large general population over time. This limitation, together with others including ascertainment and information bias, and potential bias due to missing data, are acknowledged and shared in common with other large population studies and database analyses.^29 30^ We used well-established coded definitions of outcomes and diagnoses, with two clinicians reviewing all definitions. However, we acknowledge the lack of formal adjudication of recorded outcomes and diagnosis, which was not possible given that databases are anonymised for research. Finally, alternative methods of analysis such as the use of instrumental variables to proxy unmeasured variables such as medication adherence, which our analysis could not account for, could be considered within a mediation analysis framework.

## C**onclusion**

Over half of the patients in the large general population studied did not experience an optimal reduction in their LDL-C, 24 months after starting statin therapy. These patients had a significantly increased risk of future CVD (coronary artery disease, stroke/TIA, PVD) compared with those with an optimal cholesterol response.

**Key messages**

**What is already known on this subject?**

- Statins, the most widely prescribed class of drugs, are associated with variations in cholesterol response.

**What might this study add?**

- This study provides ‘real world evidence’ that 50% of patients started on statins do not derive the intended therapeutic benefit from them, significantly increasing their risk of future cardiovascular disease.

**How might this impact on clinical practice?**

- These findings contribute to the debate on the effectiveness of statin therapy and highlight the need for personalised medicine in lipid management for patients.
