# Supplementary material for: Sub-optimal cholesterol response to initiation of statins and future risk of cardiovascular disease
Source: Heart. 2019 Apr 15;105(13):975–81. doi: 10.1136/heartjnl-2018-314253 (PMC6582718; doi:10.1136/heartjnl-2018-314253)
Supplement: Supplementary file 1 [file heartjnl-2018-314253supp001.pdf]

# ONLINE SUPPLEMENTARY

## Sub-optimal cholesterol response to initiation of statins and future risk of cardiovascular disease

**Appendix 1. List of all potential covariates assessed at baseline**

- Age at study entry (baseline)
- Gender (male or female)
- LDL cholesterol (baseline)
- Body mass index (BMI)
- Systolic blood pressure (single most recent value at baseline)
- Diastolic blood pressure (single most recent value at baseline)
- Alcohol misuse
- Smoking status (non-smoker; ex-smoker; smoker; unknown)
- Atrial fibrillation
- Chronic kidney disease
- Diabetes (using a Read codes diagnosis of diabetes and HBA1C reference ranges documented in the clinical record: non-diabetic; poorly-controlled diabetic; well-controlled diabetic; diabetic – control status unknown)
- History of dyslipidaemia
- Family history of CVD
- Family history of hyperlipidaemia
- Treated hypertension (diagnosis of hypertension and a treatment with at least one antihypertensive medication within 12 months prior to entry date)
- Hypothyroidism
- Liver disease
- Migraine
- Nephrotic syndrome
- Rheumatoid arthritis
- Severe mental illness
- Systemic lupus erythematosus
- Medication count (at baseline – within 12 months prior to entry date)
- Prescription of other lipid lowering medications (Including fibrates, bile sequestrants, nicotinic acid at baseline – within 12 months prior to entry date)
- Prescription of corticosteroids at baseline – within 12 months prior to entry date)
- Prescription of antipsychotics at baseline – within 12 months prior to entry date)
- Potency of initial prescribed statin (low; medium, high)
- Change in statin potency
- Time (year) of statin therapy initiation
- HDL cholesterol (baseline)
- Number of LDL-C measurements recorded within exposure period (24 months after statin therapy initiation).

**Appendix 2. Characteristics of eligible patients being treated with statins and incident cardiovascular disease within 24 months of initiating statin therapy (n=23,947)**

| **Characteristics** | | **Total number (%)** | **Optimal statin responders, n (%)** | **Sub-optimal statin responders, n (%)** |
|---|---|---|---|---|
| | | **23,947 (100)** | **11,800 (49.3)** | **12,147 (50.7)** |
| Follow-up time (days) | Median (IQR) | 133 (40 – 340) | 135 (41 – 337) | 130 (38 – 342) |
| Females | No. (%) | 8,968 (37.5) | 4,437 (37.6) | 4,531 (37.3) |
| Age (years) | Mean (SD) | 66.5 (11.0) | 66.9 (10.9) | 66.2 (11.0) |
| Baseline LDL cholesterol (mmol/L) | Mean (SD) | 3.7 (1.2) | 4.1 (1.0) | 3.3 (1.1) |
| Post-treatment LDL cholesterol within 24 months (mmol/L) | Mean (SD) | 2.3 (0.9) | 1.9 (0.6) | 2.7 (1.0) |
| Body mass index (kg/m2) | Mean (SD) | 28.2 (5.2) | 28.1 (5.0) | 28.4 (5.3) |
| Alcohol misuse | No. (%) | 180 (0.8) | 67 (0.6) | 113 (0.9) |
| Smoking | | | | |
| Non-smoker | No. (%) | 344 (1.4) | 154 (1.3) | 190 (1.6) |
| Ex-smoker | | 378 (1.6) | 152 (1.3) | 226 (1.9) |
| Smoker | | 344 (1.4) | 146 (1.2) | 198 (1.6) |
| Unknown status | | 22,816 (95.5) | 11,339 (96.2) | 11,477 (94.9) |
| Atrial fibrillation | No. (%) | 1,023 (4.3) | 510 (4.3) | 513 (4.2) |
| Chronic kidney disease | No. (%) | 635 (2.7) | 301 (2.6) | 334 (2.8) |
| Dyslipidaemias | No. (%) | 1,657 (6.9) | 767 (6.5) | 890 (7.3) |
| Potency of initial statin prescribed | | | | |
| Low | No. (%) | 6,306 (26.4) | 2,577 (21.8) | 3,729 (30.7) |
| Medium | | 15,425 (64.4) | 8,166 (69.2) | 7,259 (59.8) |
| High | | 2,205 (9.2) | 1,057 (9.0) | 1,148 (9.5) |

**Appendix 3. Characteristics of eligible patients being treated with statins and free from cardiovascular disease at baseline.** Patients are stratified by year of initiation of statin therapy

| | 2000 or earlier | 2001 – 2005 | 2006 – 2010 | 2011 - 2016 | p-value |
|---|---|---|---|---|---|
| | **4,201 (2.54)** | **66,944 (40.47)** | **67,751 (40.96)** | **26,515 (16.03)** | |
| Follow-up time (years), median (IQR) | 7.7 (3.4-13.9) | 8.8 (4.7-11.4) | 6.4 (4.3-8.2) | 3.0 (1.90–4.0) | 0.0001 |
| Females | 2,072 (49.3) | 34,371 (51.3) | 31,785 (46.9) | 12,142 (45.8) | <0.0001 |
| Age (years), Mean (SD) | 62.4 (10.1) | 64.2 (11.6) | 61.5 (11.7) | 60.1 (12.0) | 0.0001 |
| Baseline LDL cholesterol (mmol/L), Mean (SD) | 4.7 (1.1) | 4.1 (1.1) | 4.1 (1.1) | 4.0 (1.2) | 0.0001 |
| Body mass index (kg/m2), Mean (SD) | 28.2 (4.7) | 28.9 (5.5) | 29.2 (5.8) | 29.5 (6.0) | <0.0001 |
| Alcohol misuse | 10 (0.2) | 249 (0.4) | 503 (0.7) | 469 (1.8) | <0.0001 |
| Smoking | | | | | <0.0001 |
| Non-smoker | 32 (0.8) | 1,068 (1.6) | 1,026 (1.5) | 435 (1.6) | |
| Ex-smoker | 21 (0.5) | 758 (1.1) | 741 (1.1) | 318 (1.2) | |
| Smoker | 23 (0.6) | 719 (1.1) | 882 (1.3) | 379 (1.4) | |
| Unknown status | 4,125 (98.2) | 64,399 (96.2) | 65,102 (96.1) | 25,383 (95.7) | |
| Index of Multiple Deprivation (patients) | | | | | <0.0001 |
| 1 | 601 (23.8) | 8,979 (24.0) | 9,417 (23.1) | 2,948 (19.6) | |
| 2 | 575 (22.8) | 8,362 (22.3) | 9,012 (22.2) | 3,193 (21.2) | |
| 3 | 516 (20.5) | 7,622 (20.3) | 8,057 (19.8) | 3,029 (20.1) | |
| 4 | 454 (18.0) | 6,929 (18.5) | 7,678 (18.9) | 3,125 (20.7) | |
| 5 | 375 (14.9) | 5,601 (14.9) | 6,529 (16.0) | 2,786 (18.5) | |
| Atrial fibrillation | 103 (2.5) | 2,224 (3.3) | 1,836 (2.7) | 686 (2.6) | <0.0001 |
| Chronic kidney disease | 12 (0.3) | 357 (0.5) | 3,114 (4.6) | 986 (3.7) | <0.0001 |
| Diabetes | | | | | <0.0001 |
| Non diabetic | 3,739 (89.0) | 54,662 (81.7) | 57,663 (85.1) | 21,920 (82.7) | |
| Poorly-controlled diabetic | 106 (2.5) | 3,586 (5.4) | 3,649 (5.4) | 1,905 (7.2) | |
| Well- controlled diabetic | 70 (1.7) | 1,528 (2.3) | 1,304 (1.9) | 351 (1.3) | |
| Diabetic - control status unknown | 286 (6.8) | 7,168 (10.7) | 5,135 (7.6) | 2,339 (8.8) | |
| Dyslipidaemias | 776 (18.5) | 5,579 (8.3) | 5,063 (7.5) | 2,027 (7.6) | <0.0001 |
| Potency of initial statin prescribed | | | | | <0.0001 |
| Low | 2,679 (63.8) | 21,391 (32.0) | 12,693 (18.7) | 2,580 (9.7) | |
| Medium | 1,449 (34.5) | 42,042 (62.8) | 53,078 (78.3) | 20,695 (78.1) | |
| High | 73 (1.7) | 3,511 (5.2) | 1,982 (2.9) | 3,240 (12.2) | |

**Appendix 4. Effect estimates for association between sub-optimal LDL-C response at 24 months to initiated statin therapy and the risk of incident cardiovascular events using different statistical approaches.** Patients with post-treatment LDL-C after 3 months of statin initiation (n=149,781).

| | Group | Number of CVD events | Rate of CVD events (per 1,000 person-years) | Crude/unadjusted models | | Adjusted models | |
|---|---|---|---|---|---|---|---|
| | | | | Cox regression | Competing-risks survival regression | Cox regression | Competing-risks survival regression |
| | | | | HR (95% CI) | sHR (95% CI) | HR (95% CI) | sHR (95% CI) |
| **Overall CVD-related event** * | Optimal | 9821 | 19.9 | 1 | 1 | 1 | 1 |
| | Sub-optimal | 11387 | 22.7 | 1.16 (1.13 – 1.19) | 1.12 (1.09 – 1.15) | 1.21 (1.17 – 1.24) | 1.18 (1.15 – 1.21) ‡ |
| Coronary artery disease † | Optimal | 5557 | 11.3 | 1 | 1 | 1 | 1 |
| | Sub-optimal | 6739 | 13.4 | 1.21 (1.17 – 1.25) | 1.16 (1.12 – 1.21) | 1.28 (1.24 – 1.33) | 1.22 (1.18 – 1.26) § |
| Stroke/TIA * | Optimal | 2174 | 4.4 | 1 | 1 | 1 | 1 |
| | Sub-optimal | 2317 | 4.6 | 1.08 (1.01 – 1.14) | 1.02 (0.96 – 1.08) | 1.16 (1.09 – 1.23) | 1.11 (1.04 – 1.18) \|\| |
| Peripheral vascular disease * | Optimal | 1345 | 2.7 | 1 | 1 | 1 | 1 |
| | Sub-optimal | 1523 | 3.0 | 1.13 (1.05 – 1.21) | 1.08 (1.00 – 1.16) | 1.15 (1.06 – 1.24) | 1.10 (1.02 – 1.19) # |
| CVD-related death * | Optimal | 745 | 1.5 | 1 | 1 | 1 | 1 |
| | Sub-optimal | 808 | 1.6 | 1.09 (0.98 – 1.20) | 1.04 (0.94 – 1.15) | 1.25 (1.12 – 1.38) | 1.20 (1.08 – 1.33) ** |

Cox regression provides hazard ratio whereas competing-risks survival regression (Fine-Gray Model) provides sub-hazard ratio

* Age and baseline LDL-cholesterol level were adjusted for in the multivariable models (both Cox and competing-risks) for overall CVD-related events, stroke/TIA, PVD, and CVD-related deaths.

† Age was adjusted for in the multivariable model (both Cox and competing-risks) for coronary artery disease.

‡ Competing risks for overall CVD-related event were non-CVD-related death and transfer out of practice.

§ Competing risks for coronary artery disease model were death, transfer out of practice, stroke/TIA and PVD.

|| Competing risks for stroke/TIA model were death, transfer out of practice, coronary artery disease and PVD.

# Competing risks for PVD model were death, transfer out of practice, coronary artery disease, and stroke/TIA.

** Competing risks for CVD-related death were non-CVD-related death, transfer out of practice, coronary artery disease, stroke/TIA and PVD.

LDL-C - Low-density lipoprotein cholesterol; CPRD - Clinical Practice Research Datalink; HES - Hospital Episodes Statistics; ONS - Office of National Statistics; CVD - Cardiovascular disease; PVD – Peripheral vascular disease; TIA - transient ischaemic attack; HR - hazard ratio; CI - confidence interval; sHR - sub-hazard ratio.

**Appendix 5. Association between LDL-C reduction within 24 months of statin therapy initiation and risk of cardiovascular disease.** Patients with reduction in LDL-C within 24 months of initiating statin therapy (n=146,355)

| | **Optimal response (n=80,493)** Odds Ratio (95% CI) | **Sub-optimal response (n=65,862)** Odds Ratio (95% CI) |
|---|---|---|
| **Overall CVD event** | 0.87 (0.84 – 0.90)* | 0.94 (0.91 – 0.98)* |
| Coronary artery disease | 0.91 (0.88 – 0.95)* | 0.96 (0.92 – 1.01) |
| Stroke/TIA | 0.82 (0.77 – 0.87)* | 0.87 (0.80 – 0.95)* |
| Peripheral vascular disease | 0.87 (0.80 – 0.94)* | 1.03 (0.94 – 1.14) |
| CVD-related death | 0.88 (0.79 – 0.98)* | 0.90 (079 – 1.03) |

* Statistically significant association; LDL-C - Low-density lipoprotein cholesterol; CI – confidence interval; CVD – cardiovascular disease; TIA – transient ischaemic attack
